# Isolation and characterization of bacteriocinogenic lactic bacteria from M-Tuba and Tepache, two traditional fermented beverages in México

**DOI:** 10.1002/fsn3.236

**Published:** 2015-04-29

**Authors:** Norma M de la Fuente-Salcido, José Cristobal Castañeda-Ramírez, Blanca E García-Almendárez, Dennis K Bideshi, Rubén Salcedo-Hernández, José E Barboza-Corona

**Affiliations:** 1 Escuela de Ciencias Biológicas, Universidad Autónoma de Coahuila, Ciudad Universitaria Campus TorreónTorreón Coah, Coahuila, CP 27104, México; 2 Universidad Tecnológica del Suroeste de GuanajuatoValle de Santiago, Guanajuato, 38400, México; 3 Facultad de Química, Universidad Autónoma de Querétaro, DIPA, PROPAC76010, Santiago de Querétaro, Querétaro, Mexico; 4 Department of Natural and Mathematical Sciences, California Baptist University8432 Magnolia Avenue, Riverside, 92504, California; 5 Department of Entomology, University of CaliforniaRiverside, 92521, California; 6 Departamento de Alimentos, División de Ciencias de la Vida, Universidad de GuanajuatoIrapuato, Guanajuato, 36500, México; 7 Posgrado en Biociencias, División de Ciencias de la Vida, Universidad de GuanajuatoIrapuato, Guanajuato, 36500, México

**Keywords:** Bacteriocins, lactic acid bacteria, M-Tuba, Tepache

## Abstract

Mexican Tuba (M-Tuba) and Tepache are Mexican fermented beverages prepared mainly with pineapple pulp and coconut palm, respectively. At present, reports on the microbiota and nutritional effects of both beverages are lacking. The purpose of this study was to determine whether M-Tuba and Tepache contain cultivable lactic acid bacteria (LAB) capable of producing bacteriocins. Tepache and M-Tuba contain mesophilic aerobic bacteria, LAB, and yeast. *Bacillus subtilis*, *Listeria monocytogenes*, *Listeria innocua*, *Streptococcus agalactiae*, *Staphylococcus aureus*, *Escherichia coli*, *Klebsiella pneumoniae*, *Salmonella typhimurium*, and *Salmonella spp*, were the microorganisms most susceptible to metabolites produced by bacterial isolates. M-Tuba and Tepache contain bacteria that harbor genes coding for nisin and enterocin, but not pediocin. The presence of *Lactococcus lactis* and *E. faecium* in M-Tuba and Tepache, was identified by 16S rDNA. These bacteria produced bacteriocins of ∼3.5 kDa and 4.0–4.5 kDa, respectively. Partial purified bacteriocins showed inhibitory effect against *Micrococcus luteus*, *L. monocytogenes*, *L. innocua*, *Str. agalactiae*, *S. aureus*, *Bacillus cereus*, *B. subtilis*, *E. faecalis*, and *K. pneumoniae*. We characterized, for the first time, cultivable microbiota of M-Tuba and Tepache, and specifically, identified candidate lactic bacteria (LAB) present in these beverages that were capable of synthesizing antimicrobial peptides, which collectively could provide food preservative functions.

## Introduction

Bacteriocins are antimicrobial peptides ribosomally synthesized by diverse bacteria. These peptides constitute a type of microbial arsenal secreted by bacteria to inhibit growth of competitive microbes, including other prokaryotes, fungi, and parasites (Muñoz-Rojas 2004; Dufour et al. 2007; De la Fuente-Salcido et al. 2013). Several of these antimicrobial peptides show activity at high temperature and a wide range of pH, but can be inactivated by gut proteases. As such, these properties make bacteriocins suitable and attractive for use as food biopreservatives (Stiles 1996; Cotter et al. 2005; Gálvez et al. 2007; De la Fuente Salcido and Barboza-Corona 2010; Udhayashree et al. 2012). In particular, lactic acid bacteria (LAB) are present in the microbiota of different foods such as meat, cheese, and yogurt, and are considered as probiotics. They have been consumed in different fermented products for millennia without reports of adverse health effects. LAB synthesize metabolites that not only contribute to distinctive food flavors but importantly also produce bacteriocin preservatives that are recognized as safe by regulatory agencies, including the Food and Drug Administration (FDA) and the European Food Safety Authority (EFSA) (Millette et al. 2008; EFSA Panel on Biological Hazards (BIOHAZ) 2010; Halasz 2009).

A wide variety of distilled (tequila, stool, mezcal, bacanora) and nondistilled (pulque, tesgüino, tepache, colonche, pozol, tuba, and axokot) products are produced in Mexico (Blomberg 2000; Guyot et al. 2003; Escalante et al. 2004; Hui and Özgül Evranuz 2012). These fermented beverages have been consumed for many years and are strongly connected to the Mexican culture and ancient traditions. In particular, “Tepache,” consumed since the pre-Hispanic age, is prepared mainly with pinapple pulp, and also with maize, apple, and orange (Battcock and Azam-Ali 1998; Corona-González et al. 2006). “Tuba” is another beverage made from coconut palm, and is frequently consumed mixed with nuts, primarily in coastal regions of the States of Colima, Michoacan, and Guerrero. Tuba is prepared using the sap (nectar or honey water) collected from the inflorescence of coconut palms (Velázquez-Monreal et al. 2011; Chandrasekhar et al. 2012). Interestingly, a fermented beverage also called “Tuba,” obtained from exudate of tapped unopened spathe of coconut, is consumed not only in Mexico but also in the Philippines and other countries (Kadere and Kutima 2012). Similar Tuba products have different names worldwide, for example, Toddy, Tuak, Legmi, Mnazi, Emu, Kallu in Sri Lanka, Indonesia, Tunia, Kenyae, Nigeria, and India, respectively (Chandrasekhar et al. 2012).

The presence of bacteriocin-producing bacteria in these fermented beverages is of particular importance not only because they protect fermented foods from microbial contamination and spoilage and are responsible for flavor and aroma but also because they indirectly protect consumers from potential pathogenic bacteria. At present, reports on the microbiota and nutritional effects of Mexican Tuba and Tepache are lacking. In this regard, the purpose of this study was to determine whether these two beverages contain cultivable bacteria capable of producing bacteriocins. We demonstrated that potential probiotic and bacteriocin-producing LAB such as *L. lactis* TuAB1, *L. lactis* TeA1, *E. faecium* TuAB2, and *E. faecium* TeA2 constitute part of the cultivable microbiota of Tepache and M- Tuba that potentially elaborate inhibitory effects against pathogenic bacteria of clinical significance.

## Material and Methods

### Sample collection and bacterial strains

Mexican Tuba (M-Tuba hereafter to differentiate Tuba produced in other countries) and Tepache were obtained from three different artisan producers in Colima and Guanajuato, respectively. Samples were transported in coolers immediately for microbiological and biochemical analysis. Strains used as positive control for polymerase chain reaction assays were *Lactococcus lactis* subsp *lactis* ATCC 19435 (Microbial Culture Collection, CINVESTAV México DF), *Enterococcus faecium* UQ1 (Universidad Autónoma de Querétaro), *Enterococcus faecalis* ATCC 10541, and *Pediococcus acidilactici* (Instituto Tecnológico de Veracruz, México). These strains produce nisin, enterocin, and pediocin, respectively. Microorganisms used as indicator bacteria for the overlay method or diffusion assay were *Listeria innocua*, *L. monocytogenes*, *Staphylococcus aureus* ATCC 25923, *Streptococcus agalactiae*, *Bacillus subtilis* ATCC 6633, *Enterococcus faecalis* ATCC 10541, *Escherichia coli* ATCC 25932, *Salmonella* spp *S. typhimurium* ATCC 14022, and *Klebsiella pneumoniae*. Bacteria were maintained as frozen stocks at −20°C in 30% (v/v) glycerol and routinely cultivated at 35 ± 2°C on MRS (DIFCO) or TSB (trypticase soy broth) (BD) for PCR test or detection of antimicrobial activity.

### Standard count, isolation, and preliminary biochemical identification of LAB

Ten mL of the fermented beverages were added to 90 mL of sterile saline solution (0.85% NaCl) (w/v), and then serially diluted (10^−1^ to 10^−6^). From each dilution, duplicate 0.1-mL samples were innoculated in different culture media: (1) Standard methods agar (BD Bioxon, Cuahutitlán Izcalli, Estado de México, México) at 35 ± 2°C, 48 h for mesophilic bacteria count; (2) MRS agar at 30°C for 48 h under anaerobic conditions (BD GasPak EZ Anaerobe Container System) for detection of lactic bacteria (LAB); and (3) potato dextrose agar (PDA) (BD Bioxon) plus chloramphenicol (0.004 mg L^−1^), at 25°C for 48 h for yeast (NOM-142-SSA1-1995). LAB isolation was performed using two media, that is, MRS plus glucose (10%) and peptonized milk agar (BD Bioxon). Plates were incubated at 35 ± 2°C in aerobic and anaerobic conditions using a BD GasPak EZ Anaerobe Container System (BD Bioxon, Cuahutitlán Izcalli, Estado de México, México) for 48 h. Isolates were stored in either nutrient agar plates at 4°C or MRS broth-sterile glycerol (30% v/v) for long-term storage at −70°C. Once bacteria were isolated they were preliminarily identified by Gram-staining, colonial, and cellular morphology. Simultaneously, several physiological and physical characteristics were assessed, including catalase and oxidase activities, carbohydrate (glucose, lactose, sucrose) fermentation with gas production, carbon dioxide (CO_2_) production from glucose, amines from arginine, motility, and the ability of isolates to grow at different temperatures, pH, and salt concentration. All tests were performed according to the established protocols (Tang et al. 1998; Salminen et al. 2004; Barbu 2008).

### Detection of bacteriocin-producing bacteria by the overlay method

Indicator (sensitive) strains were grown overnight in MRS broth, nutrient broth, brain-heart infusion broth, Luria broth, TSB, or tetrathionate broth with orbital shaking (180 rpm) at optimal temperature (28 or 35 ± 2°C) to a cell density of 10^8^–10^9^ cell/mL. Potential LAB isolates were cultured for 24–48 h in MRS broth (15 mL) incubated in both aerobic and anaerobic conditions with orbital shaking. These isolates were also inoculated in duplicate parallel streaks (1 cm) or drops (10 *μ*L) on MRS agar plates and incubated overnight to detect optimal growth at 35 ± 2°C. One of the MRS plates was overlaid with TSB soft agar (0.75%) containing 105 *μ*L (0.7%) of each indicator strains (∼10^8^ cell/mL) and incubated at 35 ± 2°C for 24 h. The other plate was used to select bacteriocinogenic colonies that showed inhibitory effects in the overlay assay (Barboza-Corona et al. 2007).

### Screening of bacteriocin genes by multiplex polymerase chain reaction (mPCR)

Bacteria with characteristics of LAB that showed inhibitory activity against sensitive strains were used for screening bacteriocins genes by mPCR. Pools of bacteria were cultivated in MRS broth for 36 to 48 h. Then 1.5 mL of each culture was centrifuge (16,000 ×*g*), and pellets were resuspended in 200 *μ*L of 10 mmol/L Tris, pH 8.0. Samples were incubated at −20°C for 30 min, then for 10 min in boiling water and centrifuged to remove cellular debris (Barboza-Corona et al. 2007). DNA present in the supernatants was used as template for amplifying conserved regions of nisin (class I), enterocin, and pediocin (class II) genes. Amplicons were obtained by mPCR using the Taq pol (Invitrogen, Carlsbad, CA) and a mix of specific bacteriocins oligonucleotides (Table1) previously reported (Suwanjinda et al. 2007; Wieckowicz et al. 2011). Amplification was performed in a C1000 Touch Thermal Cycler (Bio-Rad, Hercules, CA) for 30 cycles as follows: 95°C for 30 sec; 45–55°C for 45 sec, 68°C for 45 sec, followed by a 4-min termination cycle at 68°C. Amplicon sizes were compared to those obtained from LAB that synthesize nisin, enterocin, and pediocin, respectively, *Lactococcus lactis*, *Enterococcus faecium*, and *Pediococcus acidilactici*. In addition, amplicons of the expected size were cloned into the pCR 4-TOPO vector (Invitrogen) and the ligated product was transformed in *E. coli* DH5*α*F cells using an *E. coli* pulser (BioRad). Transformant was selected, and recombinant plasmid was purified using the QIAquick gel extraction kit (Qiagen, Valencia, CA), and submitted for sequencing to the National Laboratory of Genomics for Biodiversity (Langebio, at CINVESTAV-Irapuato, México), sequences were compared to those reported in the NCBI databases (www.ncbi.nlm.nih.gov).

**Table 1:** Specific primers used for the PCR detection of bacteriocin genes^1^

Bacteriocin	Forward (F) and reverse (R) primers	Amplicon size (bp)
Nisin	nisRF 5’-CTATGAAGTTGCGACGCATCA-3’	608
	nisRR 5’-CATGCCACTGATACCCAAGT-3	
Enterocin	entF 5’-GGGTACCACTCATAGTGGAA-3’	412
	entR 5’-CCAGCAGTTCTTCCAATTTCA-3’	
Pediocin	pedF 5’-GGTAAGGCTACCACTTGCAT-3’	332
	pedR 5’-CTACTAACGCTTGGCTGGCA-3’	

1 nis, nisin; ent, enterocin; ped, pediocin. Sequences were taken from Suwanjinda et al. (2007).

### Identification of bacteriocinogenic bacteria

Bacteria in each group that showed amplification of bacteriocin genes was analyzed individually. Identification to genus and species level was determined using the API 50 CH system (API systems, BioMéreux, Boston, MA, USA) and by the amplification of 16S rDNA using the universal oligonucleotide pair that amplified both the bacterial and archaeal domains: forward UBF 5′-AGAGTTTGATCCTGGCTGAG-3′ and reverse 1492 R5′-GGTTACCTTGTTACGACTT-3′ (León-Galván et al. 2009). For amplification of 16S rDNA, a high-fidelity enzyme (BioRad) was used under the following conditions: 5 min at 95°C; 30 cycles of 30 sec at 95°C, 30 sec at 55°C, and 1:40 min at 68°C; and finally 5 min at 72°C.

### Production, determination of molecular size and activity of bacteriocins

The production of bacteriocins from selected strains was carried out in MRS broth inoculated with a preculture of each strain (10^9^ cells/mL) grown overnight in the same broth at 35 ± 2°C and 180 rpm. After 24 h, each culture broth was centrifugated at 10, 000 × *g* for 15 min, precipitated with ammonium sulfate (80% saturation) at 4°C, resuspended in 100 mmol/L phosphate buffer (pH 6.8), and dialyzed overnight using a 1-kDa cutoff membrane (Amersham Biosciences Corp., Piscataway, NJ, USA). Dialyzed crude extracts were treated separately with proteinase K (NEB) at a final concentration of 1 mg/mL. Untreated extract plus buffer, buffer without extract and enzyme solutions were used as controls. All the reactions were incubated at 42°C for 2 h at the pH recommended by the suppliers. Mixtures were heated at 100°C for 10 min to stop enzyme reaction. Antibacterial activity after enzyme treatment was determined using the modified well diffusion method (Barboza-Corona et al. 2007). Briefly, ∼25 mL of assay agar plus 2% tween 20 (v/v) or TSB with soft agar 0.7% (w/v) were mixed with 75 *μ*L of each indicator strain (*M. luteus*, *L. innocua*, *L. monocytogenes*, *E. faecalis*) and then poured into petri dishes. Wells, 8 mm in diameter, were dug into the agar and 100 *μ*L of the bacteriocins extracts were added to each well and incubated for 12 h at 4°C, followed by an additional incubation at 28°C or 35 ± 2°C for 24–48 h. The diameters of zones of inhibition were measured. Assays were repeated in triplicate and the average was recorded. One unit of extract activity was defined to be equal to 1 mm^2^ of the zone of inhibition of growth of the indicator bacterium (Barboza-Corona et al. 2007). The antibacterial activity was determined against strains used in overlay method (*Str. agalactiae*, *S. aureus*, *B. subtilis*, *K. pneumonia*, *E. coli*, *Salmonella* spp, and *S. typhimurium*). The molecular masses of bacteriocins were determined by direct detection in a polyacrylamide gel overlay assay. Samples were fractionated in duplicated in a 16% sodium dodecylsulfate (SDS)-polyacrylamide gel (SDS-PAGE) (Schägger and Von Jagow 1987). Then one gel was stained with brilliant blue G (Serva) and the other was used for direct detection of antibacterial activity. This second gel was fixed in 20% (v/v) isopropanol and 10% (v/v) acetic acid at room temperature for 0.5 h and washed with sterile distilled water for 1 h. Finally, the gel was overlaid with TSB soft agar supplemented with 1% (v/v) of broth cultures of different indicator bacteria and then incubated overnight at 28°C or 35 ± 2°C.

## Results

### Total count, biochemical properties, and growth of bacterial isolates under different physical conditions

A screen of the microbiota present in M-Tuba and Tepache capable of growth in MRS, and determination of the total number of mesophilic and lactic acid bacteria (LAB) and yeasts were carried out according to the Mexican Official Norms (NOM-142-SSA1-1995, www.dof.gob.mx/index.php) for the fermented beverages. Tepache contained 7.992, 5.077 and 4.984 log CFU/mL, whereas M-Tuba harbored 6.948, 4.967, and 5.10 log CFU/mL of mesophilic aerobic bacteria, LAB, and yeast, respectively. From these, 158 bacterial isolates were further studied. When isolates were grown in MRS agar, bacteria colonies were predominantly small, convex, white, circular, and with rounded edges. Of these, 74% of the isolates were cocci, of which 53% gram-positive, 68% catalase-negative, 84% oxidase negative, and 90% were nonspore-forming bacteria (Table2).

**Table 2:** Biochemical properties and biophysical properties of bacteria isolated from Tepache and M-Tuba

Characteristic	% of total bacterial isolated^1^
Gram staining (+)	53 (+), 47 (−)
Catalase activity	16 (+), 68 (−), 16 (±)
Oxidase activity	80 (+), 16 (−), 4 (±)
Glucose	43 (+), 57 (−)
Lactose	22 (+), 78 (−)
Sucrose	16 (+), 84 (−)
Production of NH_3_ from arginine^2^	96 (+), 4 (±)
Growth at 15°C	100 (+)
Growth at 45°C	87 (+), 13 (−)
Growth at pH 4	64 (+), 36 (−)
Growth at pH 5	90 (+), 10 (−)
Growth at pH 6.5	59 (+), 41 (−)
Growth in medium with NaCl (4.0%)	74 (+), 16 (−)
Growth in medium with NaCl (6.5%)	68 (+), 10 (−), 22 (±)
CO_2_ production	43 (+), 57 (−)
Citrate hydrolysis	5 (+), 95 (−)
Motility	38 (+), 62 (−)
Spore forming	10 (±), 90 (−)

(+) Positive reaction, (−) Negative Reaction, (±) Variable reaction.

1 The % is based on 158 isolated strains.

2 Nessler test.

### Detection of bacteriocin-producing bacteria using the overlay method

Gram-positive isolates were tested for their ability to inhibit the growth of different bacteria using the overlay method. We assayed the inhibitory activity against five gram-positive (*Listeria innocua*, *L. monocytogenes*, *Staphylococcus aureus*, *Streptococcus agalactiae*, *Bacillus subtilis*) and four gram-negative (*Escherichia coli*, *Salmonella spp*. *Salmonella typhimurium*, *Klebsiella pneumoniae*) indicator bacteria (Table3). We observed that both fermented beverages contained isolates with inhibitory activity against indicator bacteria tested by the overlay protocol. The most susceptible bacterium was *B. subtilis* followed by *L. monocytogenes*, *E. coli*, *L. inoccua*, *K. pneomoniae*, *S. typhimurium*, *Str. agalactiae*, *S. aureus*, and *Salmonella* spp., (Table3).

**Table 3:** Detection of bacterial isolates from M-Tuba and Tepache with inhibitory activity

Culture conditions (Medium/temperature °C)	Sensitive strains	Bacterial isolates		
		M-Tuba	Tepache	Total
MRS Broth/35 ± 2	*L. monocytogenes*	43	49	92
MRS Broth/35 ± 2	*L. innocua*	46	42	88
NB/BHI/35 ± 2	*Str. agalactiae*	57	19	76
NB/BHI/35 ± 2	*S. aureus*	41	35	76
LB/TSB/28	*B. subtilis*	51	46	97
BHI/35 ± 2	*K. pneumoniae*	79	6	85
LB/35 ± 2	*Escherichia coli*	66	23	89
TB/BHI/35 ± 2	*Salmonella spp*	36	36	72
TB/BHI/35 ± 2	*S. typhimurium*	79	6	85

MRS Broth, Man Rogosa Sharpe broth; NB, Nutrient broth; BHI, Brain-heart infusion broth; LB, Luria broth; TSB, trypticase soy broth; TB, tetrathionate broth.

### Screening of bacteriocin genes in LAB isolates from fermented beverages

**Figure 1:**
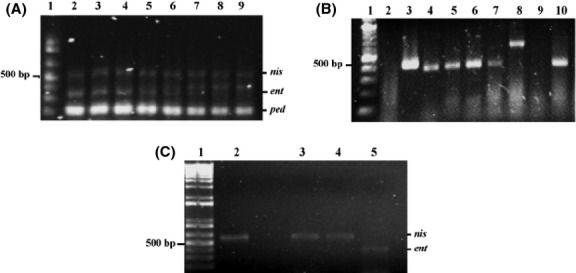
Detection of nisin, enterocin, and pediocin genes in different bacterial strains isolated from M-Tuba and Tepache. (A) Standardization of conditions for multiplex PCR (mPCR). Mixes DNA samples from *Lactococcus lactis*,*Enterococcus faecium*, and *Pediococcus acidilactici* were used as positive controls to amplify nisin, enterocin, and pediocin, respectively. Lane 1, DNA Ladder 1 Kb Plus DNA (Invitrogen); lanes 2–9, amplicons obtained from positive bacteria using an alignment interval of temperature of 48.0 to 57.5°C (intervals of 57.5, 56.8, 54.5, 55.7, 53.9, 51.5, 49.9, 48.7, and 48.0 respectively). For further amplification, we select the alignment temperatures of 54.5°C, which correspond to the amplification in lane 4. (B) Multiplex PCR of isolates obtained from Tepache and M-Tuba. Lane 1, DNA Ladder 100 bp (NEB); lanes 2–7 and 10, amplicons corresponding to the enterocin gene (∼412 bp); lane 8, nisin (∼ 608 bp). (C) Identification of nisin and enterocin genes from different isolates. Lane 1, DNA Ladder 1 kb Plus DNA (Invitrogen); lane 2, amplicon of nisin from *L. lactis* used as positive control; lanes 3 and 4, amplicons corresponding to nisin from *L. lactis* TuAB1 and *L. lactis* TeA1; lane 5, amplicons of enterocin from *E. faecium* TuAB2. Similar amplicon than *E. faecium* TuAB2 was obtained with *E. faecium* TeA2 (data not shown). Confirmation that amplicons correspond to nisin and enterocin genes, was carried out by sequencing.

**Figure 2:**
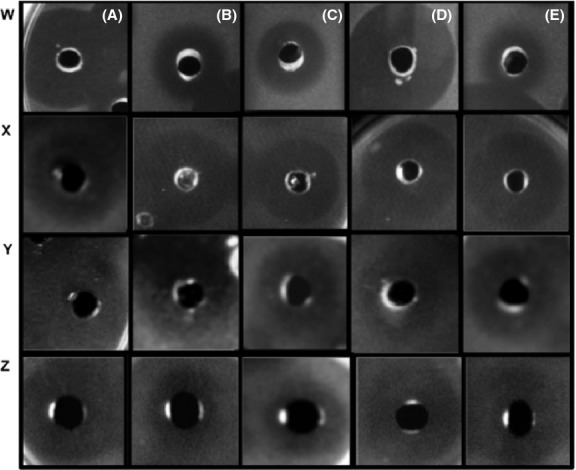
Determination of antibacterial activity by the well diffusion method of bacteriocins secreted by strains isolated from M-Tuba (Tu) and Tepache (Te). Inhibition of (W) *M. luteus*, (X) *L. monocytogenes* (Y) *L. innocua*, (Z), and *E. faecalis* using different bacteriocins. Nisin from (A) *L. lactis* used as control, (B) *L. lactis* TuAB1, and (C) *L. lactis* TeA1. Enterocin from (D) *E. faecium* UQ1 used as control and (E) *E. faecium* Tu AB2.

Lactic acid bacteria of the same fermented beverage that showed inhibitory activity were grouped in elements of tens. Groups were analyzed to determine whether they harbor putative bacteriocin genes that encode nisin, enterocin, and pediocin by mPCR. First, we standardized the condition for the mPCR using specific oligonucleotides. PCR of genes from *L. lactic*, *E. faecium*, and *P. acidilactici* produced amplicons of 0.608, 0.412, and 0.332 kbp, which correspond to the expected size of nisin, enterocin, and pediocin, respectively (Fig.1A). mPCR demonstrated that nisin and/or enterocin genes were present in all groups, but not pediocins, suggesting that M-Tuba and Tepache contained isolates capable of synthesizing nisin and enterocin (Fig.2). To detect which bacterium of each group harbor a bacteriocin gene, isolates in positive groups were analyzed individually by mPCR, and then selected for further biochemical and genetic identification. Amplicons were cloned into the pCR 4-TOPO vector (Invitrogen) and submitted for sequencing. Sequences showed ∼99% to those reported in GenBank data for nisin and enterocin.

### Bacterial identification and direct detection of bacteriocin activity in gel overlay assay

Bacteria from each group with positive amplicons for nisin and enterocin genes were identified using the API 50 CH test. Based on carbohydrate utilization patterns and amplification of the 16S rDNA, we identified two bacteria: *Lactococcus lactis* and *Enterococcus faecium*. Bacteria isolated from M-Tuba (Tu) and Tepache (Te) were named as *L. lactics* TuAB1, *E. faecium* TuAB2, and *L. lactis* TeA1, *E. faecium* TeA2, respectively. *L. lactis* and *E. faecium*, harbored nisin and enterocin genes, respectively. The inhibitory effect of the four strains was tested against different bacteria using the well diffusion method. They showed inhibitory effects against *M. luteus*, *L. monocytogenes*, *L. innocua*, *Str. agalactiae*, *S. aureus*, *B. cereus*, *B. subtilis*, *E. faecalis*, and *K. pneumoniae* (Table4).

**Table 4:** Activity of bacteriocins produced by bacteria isolated from fermented beverages

Source	Bacteria	Bacteriocin	Susceptible bacteria (units of inhibitory activity)^1^
M-Tuba	*L. lactis* TuAB1	Nisin	*M. luteus* (264), *L. monocytogenes* (63), *L. innocua* (151), *Str. agalactiae* (63), *S. aureus* (82), *B. cereus* (13), *B. subtilis* (28), *E. faecalis* (63), *K. pneumoniae* (63), *E. coli* (13)
	*E. faecium* TuAB2	Enterocin	*M. luteus* (151), *L. monocytogenes* (126), *L. innocua* (226), *Str. agalactiae* (45), *S. aureus* (63), *B. cereus* (28), *B. subtilis* (28), *E. faecalis* (126), *K. pneumoniae* (13), *E. coli* (28)
Tepache	*L. lactis* TeA1	Nisin	*M. luteus* (104), *L. monocytogenes* (151), *L. innocua* (151), *Str. agalactiae* (82), *S. aureus* (82), *B. cereus* (28), *B. subtilis* (45), *E. faecalis* (84), *K. pneumoniae* (63), *E. coli* (13)
	*E. faecium* TeA2		*M. luteus* (126), *L. monocytogenes* (82), *L. innocua* (104), *Str. agalactiae* (45), *S. aureus* (104), *B. cereus* (28), *B. subtilis* (28), *K. pneumoniae* (28)

1 In parenthesis is indicated that inhibitory activity was determined by the well diffusion method. One unit was defined as equal to 1 mm^2^ of the zone of inhibition of growth of the indicator bacterium (24).

### Determination of molecular weight and inhibitory activity of bacteriocin in crude samples

**Figure 3:**
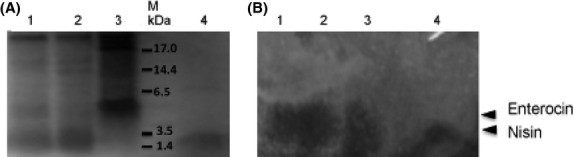
Direct detection of antibacterial activity of bacteriocins in the secretome (crude extracts) of strains isolated from M-Tuba and Tepache. *L. lactics* and *E. faecium* produce nisin and enterocin, respectively. (A) Tris-tricine SDS-PAGE. (B) Direct detection of inhibitory activity of bacteriocins isolated from M-Tuba and Tepache by gel overlay assay. Lane 1, *L. lactics* TuAB1; lane 2, *L. lactics* TeA1; lane 3, *E. faecium* TuAB2; Lane M, protein marker (BioRad); lane 4, commercial nisin used as control (Sigma). (B) Gel was overlayed with *M. luteus*. Similar results were observed when the gel was overlayed with *L. innocua* and *L. monocytogenes*. Triangles indicate the position of nisin and enterocin.

To determine the biochemical nature of partial purified bacteriocins from *L. lactis* and *E. faecium*, samples were treated with proteinase K. We observed that bacteriocin activity was abolished after treatment with proteinase K, indicating the proteinaceous nature of the bactericidal compounds. When secreted peptides of *L. lactics* TuAB1, *E. faecium* TuAB2, *L. lactis* TeA1, and *E. faecium TeA2* were subjected to Tris-tricine SDS-PAGE, species of ∼3.5 kDa and 4.0–4.5 kDa were obtained, which correspond to the molecular masses of nisin and enterocin used as controls (Fig.3).

## Discussion

Mexican food markets are continuously invaded by artificial beverages that have little or no significant nutritional value. In addition, many of these products are known to contribute to obesity and diabetes, among others diseases, which are detrimental to Mexican families (Barquera et al. 2008; Astudillo 2014). As an alternative to commercial beverages, many Mexicans in different cities and communities continue to drink traditional fermented beverages. Although traditional fermented beverages have been associated with the presence of microbial populations with some particular health benefits, there is little or no scientific information confirming the positive protective or nutritional effects of consuming these drinks (ben Omar and Ampe 2000; Sun-Waterhouse 2011; Katan 2012; Marsh et al. 2014). As no previous reports on cultivable LAB of M-Tuba and Tepache capable of synthesizing bacteriocins have been published, we were interested in characterizing the microbiota and then in further works study the uncultivable microbiota through metagenomic analysis and also the health benefits of these fermented products in order to gain insights into both the nutritional-probiotic value and microbial factors that contribute to the integrity of these products. These insights could allow for development of new fermented products using the isolated bacteria.

We found that M-Tuba and Tepache contain ∼10^5^ CFU/mL of mesophilic bacteria, and lactic bacteria (LAB) were more abundant in Tepache than in M-Tuba. In contrast, the total number of yeasts isolated from M-Tuba was greater than in Tepache. The finding of these microorganisms was in agreement with previous reports that indicate that M-Tuba fermentation process is a result of the action of multiple microorganisms including *Saccharomyces cerevisiae*, and LAB and acetic acid-producing bacteria (Stringini et al. 2009). It is expected that a similar microbiota may be present in M-Tuba and Tuba synthesized in other countries (i.e., Toddy, Tuak, Legmi, Mnazi, Emu, Kallu), but differences could result from the use of different palm and the methods used to prepare the beverage (Chandrasekhar et al. 2012; Kadere and Kutima 2012).

The presence of bacteriocin-producing *L. lactics* TuAB1 and *E. faecium* TuAB2 in M-Tuba, and *L. lactis* TeA1 and *E. faecium* TeA2 in Tepache indicate that these fermented beverages contain a natural source of potential antimicrobials that could play a role in preservation of these products. Indeed, we showed that bacteriocins genes, putatively encoding nisin and enterocin are present in, respectively, *L. lactis* and *E. faecium*. Moreover, functional peptides corresponding to these bacteriocins were observed in overlay assays.

With regard to the general effect of bacteriocins identified in the present study (i.e., nisin and enterocin), their ability to inhibit known pathogenic organism in our in vitro assays, suggests that these antimicrobial peptides may be responsible for eliminating or suppressing not only the normal microbiota present in the natural ingredients used in making M-Tuba and Tepache but also bacteria known to be etiologic agents of enteric and other diseases including systemic infections and meningitis, that is, *Staphylococcus aureus*, *Str. agalactiae*, and *Listeria species*, that could be introduced through inadvertent cross contamination. Although bacteriocins produced by LAB isolated in this study could play a primary role in suppressing or eliminating potential harmful microbes and commensals present in these natural products, it is expected that other metabolites contribute to the biochemical integrity of these products; these include small organic acids such as lactic, acetic, and formic acids, which acidify the growth medium (Broberg et al. 2007), and 2,3-butadione, reuterin, acetaldehyde, and hydrogen peroxide (Schnurer and Magnusson 2005; Pérez et al. 2014).

In future works, it will be important determine whether *L. lactics* TuAB1 and *E. faecium* TuAB2 in M-Tuba, and *L. lactis* TeA1 and *E. faecium* TeA2 in Tepache, are responsible for the typical characteristics of those beverages (i.e., aroma, correct balance of flavor, texture, acidification, etc), so that they could be used as starter strains for mass production (Amenu 2013; Marsh et al. 2014) or in the generation of new nondairy fermented beverages. In addition, the health-promoting potential of M-Tuba and Tepache could be incremented, in the interests of consumers, through the addition of certified probiotics (Marsh et al. 2014).

In conclusion, in the present study we characterized the cultivable microbiota of Mexican Tuba (M-Tuba) and Tepache, and specifically, identified candidate lactic bacteria (LAB) present in these beverages that were capable of synthesizing antimicrobial peptides, such as nisin, enterocin, and pediocin, which collectively could provide food preservative functions.
